# Early life adversity as a risk factor for cognitive impairment and Alzheimer’s disease

**DOI:** 10.1186/s40035-023-00355-z

**Published:** 2023-05-12

**Authors:** Zhihai Huang, J. Dedrick Jordan, Quanguang Zhang

**Affiliations:** Department of Neurology, Louisiana State University Health Sciences Center, 1501 Kings Highway, Shreveport, LA 71103 USA

**Keywords:** Early life adversity, Cognitive impairment, Alzheimer’s disease, Dementia, Stress

## Abstract

Neurological conditions, including cognitive impairment and Alzheimer’s disease (AD), impose a huge burden on society, affecting millions of people globally. In addition to genetic factors, recent studies indicate that environmental and experiential factors may contribute to the pathogenesis of these diseases. Early life adversity (ELA) has a profound impact on brain function and health later in life. In rodent models, exposure to ELA results in specific cognitive deficits and aggravated AD pathology. Extensive concerns have been raised regarding the higher risk of developing cognitive impairments in people with a history of ELA. In this review, we scrutinize findings from human and animal studies focusing on the connection of ELA with cognitive impairment and AD. These discoveries suggest that ELA, especially at early postnatal stages, increases susceptibility to cognitive impairment and AD later in life. In terms of mechanisms, ELA could lead to dysregulation of the hypothalamus-pituitary-adrenal axis, altered gut microbiome, persistent inflammation, oligodendrocyte dysfunction, hypomyelination, and aberrant adult hippocampal neurogenesis. Crosstalks among these events may synergistically contribute to cognitive impairment later in life. Additionally, we discuss several interventions that may alleviate adverse consequences of ELA. Further investigation into this crucial area will help improve ELA management and reduce the burden of related neurological conditions.

## Background

Cognitive impairment and Alzheimer’s disease (AD) are highly prevalent, affecting nearly 50 million people worldwide [1]. As a result, these conditions impart an outsized economic and emotional burden on individuals and society [2]. Moreover, as the populations of many nations age, the impact of neurological disorders is anticipated to rise dramatically in the coming decades [3, 4]. Several risk factors have been described to explain the pathogenesis of these diseases, including genetics, inadequate sleep, acquired factors, stress, and other environmental factors [5, 6]. Intriguingly, evidence from both human and animal research suggests that some environmental factors, particularly adverse early life experiences, may serve as key drivers in the pathogenesis of many neurological conditions [7, 8].

The developing brain is highly vulnerable to environmental factors in the first few years of life. Experiences during this time can permanently alter brain structure and function through epigenetic modifications, consequently increasing susceptibility to mental illness later in life [9–11]. Early parental caregiving has been proposed as a modulator for children’s mental development. Adequate early parental caregiving positively affects the brain development of the offspring, while poor parental caregiving could serve as a risk factor for adult mental illness in the offspring, as reviewed previously [12]. Adverse experiences in early life, including neglect and physical and emotional abuse, are known as early life adversity (ELA). Recent discoveries from both human studies and experimental animal models have supported the association between ELA and conduct disorders, impaired cognitive development, and a higher risk of dementia, AD and related neurodegenerative diseases [7, 8]. Although significant efforts have been made to mitigate the public health challenges of ELA in recent years, current understanding of this issue remains limited [13]. Hence, a better understanding of how ELA increases the risk of cognitive impairment and AD, as well as the corresponding management strategies, may help lessen the adverse consequences of ELA and reduce the global burden of these neurological illnesses.

In this review, we discuss the role of ELA in driving cognitive impairment and the onset of AD later in life. Despite the large number of cohort studies focusing on this issue, it is challenging to conduct invasive research on humans, which makes it difficult to establish a causal link. Thus, we will look at animal studies in-depth. In particular, this review will focus on potential mechanisms underlying ELA-related increased susceptibility to cognitive impairment and AD. Understanding these pathogenic processes will facilitate the development of therapeutic approaches. Finally, we summarize the current management strategies for ELA and propose potential interventions.

## Main text

### Consequences of ELA on later life health

ELA commonly involves exposure to adverse environmental circumstances during early life, such as physical, emotional, or sexual abuse and neglect [14, 15]. Nearly two decades have passed since the first discovery of strong links of adverse childhood experiences to increased lifetime risk of major diseases in a retrospective study[16]. Since then, concerns surrounding the effects of ELA on long-term health outcomes have grown substantially. Although an early report in rodents demonstrated that mild sensory stimulation in early life may favor neural connections and growth of the brain, thus providing benefits for cognitive and emotional development, chronic/extreme stress in early life may have detrimental effects on health [17]. Numerous human studies have demonstrated that adverse experiences during this sensitive period of development can increase the risk of a variety of adult diseases, including psychiatric illnesses, cardiovascular diseases, diabetes mellitus, and neurodegenerative diseases [18, 19].

ELA can also increase the risk of certain diseases in a degree-dependent manner later in life. As demonstrated in a national cross-sectional survey of adults in the US, exposure to multiple or repeated ELAs is associated with even poorer health outcomes [20]. Population-based studies suggest that the prevalence of ELA has increased to epidemic proportions, leading to a public health crisis [13, 21]. This has prompted researchers to consider the broader implications of ELA. Indeed, emerging empirical data from human and animal studies indicate that ELA exposure is connected to an increased risk of cognitive impairment in adulthood, as well as AD and other forms of dementia [7, 22–25]. Studies in animal models have shown that ELA can modulate neuronal morphology, especially causing dendritic changes [26, 27]. These results highlight the significant effects of ELA on the central nervous system (CNS), although the effects are yet to be fully understood. Moreover, to date, the link between ELA and neurodegenerative conditions has not been thoroughly investigated.

### Animal models of ELA

Multiple animal models of ELA have been developed to mimic the long-term consequences of early adverse experiences in humans [28, 29]. With these animal models, research can be conducted under controlled conditions to compensate for limitations in human studies and establish a causal connection between these variables. In most cases, animal models of ELA involve stress exposure and changing the amount and quality of parental care during the early postnatal period, as described below.

#### Maternal separation/deprivation

Maternal separation or deprivation procedures involve altering mother-pup interactions during the early postnatal period. In the maternal separation procedure, pups are separated from the dam for 2–5 h/day over a set number of days, to induce an acute predictable level of stress [30–33]. Correspondingly, maternal deprivation involves prolonged separation of dam and pups during the early postnatal period, typically a single 24-h maternal deprivation session [34]. Long-term behavioral abnormalities and impaired cognitive performance have been reported in pups exposed to these paradigms [32].

#### Limited bedding and nesting (LBN) conditions

LBN conditions are another widely used ELA paradigm to simulate resource scarcity and provoke aberrant maternal care [35–38]. In this paradigm, most bedding and nesting materials in the home cage are removed, triggering mild, chronic stress in the dam. As a result, maternal care, a crucial source of environmental sensory signals for the developing brain, can become erratic and fragmented [35, 39]. This leads to abnormalities of maternal-derived sensory input and negatively impacts long-term outcomes for pups [35, 39, 40].

#### Chronic early life stress

Paradigms that cause chronic early life stress involve exposing pups to multiple stressors or repetitive, prolonged stressors. In some paradigms, the pups are exposed to 3 to 4 distinct types of stressors, generally comprising forced swimming, physical restriction, placement on an elevated platform, and foot shock exposure in early postnatal days (PND) [41, 42]. These combined factors can lead to significant physical and psychological stress [41]. Furthermore, an early foot shock paradigm has been developed to imitate early trauma or abuse experiences [43, 44]. Briefly, the pups are put in a closed, dark, electric shock apparatus during early postnatal time windows and receive a continuous electric foot shock, which can cause severe psychological and physiological stress [45, 46].

### ELA and cognitive impairment

#### Studies in animals

Up to now, most studies on the association between ELA and cognitive outcomes have been conducted in rodents (Table 1). These animal models allow studies based on specific hypotheses and enable controlled conditions of experiments to avoid the inherent ethical issues in human studies. Preclinical animal studies have demonstrated that maternal care during early life profoundly impacts many aspects of the cognitive ability of offspring, and these effects can persist into adulthood [47, 48]. Pups that received high levels of maternal caregiving express higher levels of neurotrophic factors and exhibit better spatial learning and memory [47]. Based on these findings, many studies have been conducted in animal models to explore how inadequate maternal care influences the cognitive outcomes of the offspring. Several research groups have reported poor cognitive outcomes in adult rodents with a history of maternal separation/deprivation [22, 49–54]. Notably, these deficits are not apparent in PND-21 female animals with maternal deprivation during the early postnatal period, in comparison to age-matched male animals [55]. However, severe cognitive deficits have been observed in female animals on PND 40, indicating that the susceptibility to cognitive impairment might increase with age in female animals [49]. Likewise, using a visual-discrimination task, Yajima and colleagues [22] found that mice exposed to maternal deprivation display more evident cognitive impairment in middle age (1.4 years of age) than at a younger age. Intriguingly, lower levels of brain-derived neurotrophic factor (BDNF) and synapse-related proteins, accompanied by reduced number of mature neurons, were also detected in the hippocampus and prefrontal cortex of animals subjected to maternal separation/deprivation [49, 54]. This further supports poor maternal care as a potential risk factor for abnormal brain development.

**Table 1:** Animal studies on ELA and cognitive impairment

ELA model	Species/sex	Period of exposure	Time of outcome measurement	Cognitive outcome	References
LBN	Sprague Dawley rats/male	PND 2–9	4–5, and 12 months of age	At 12 months of age↓	[57]
Chronic stress	Wistar rats/male	PND 26–28	2 and 3 months of age	↓	[41]
LBN	Sprague Dawley rats/male	PND 2–9	10–12 months of age	↓	[58]
LBN	CRF1-CKO mice or wild type mice/male	PND 2–9	6 months of age	↓	[56]
MD	Wistar rats/male and female	PND 9	PND 40	Male↓; Female↓↓	[49]
LBN	C57Bl/6J mice	PND 2–9	5 months of age	Male↓↓; Female↓	[59]
Foot shock	Wistar rats/male	PND 21–26	Adulthood	↓	[60]
MS	Sprague Dawley rats/male and female	PND 2–21	PND 55	↓	[50]
Single prolonged stress	Sprague Dawley rats/male	PND 25	PND 32, 60 and 90	At PND 32 and 60↓	[61]
MD	Wistar rats/female	PND 3	12–17 weeks of age	↓	[51]
Foot shock	Wistar rats/male	PND 21–27	10 weeks of age	↓	[44]
MS	Balb/cJ and C57Bl/6J mice/male and female	PND 2–15	2 months of age	↓	[52]
MD	C57BL/6J mice/male	PND 2–14	2.5 months and 1.4 years of age	At 2.5 months of age↓ and 1.4 years of age↓↓	[22]
MD	Sprague Dawley rats/male and female	PND 9	PND 74	↓	[53]
MD	Sprague Dawley rats/male and female	PND 2–14	PND 21 and 25	Male↓	[55]
MD	C57BL/6 mice/female	PND 2–14	Adulthood	↓	[54]

*ELA* early life adversity; *LBN* limited bedding and nesting conditions; *MS* maternal separation; *MD* maternal deprivation; *PND* postnatal day

The symbol “↓” represents a decrease, and “↓↓” indicates a more significant decrease

Consistent with these findings, abnormal maternal behaviors, such as hypervigilance and abuse, which are induced by the LBN paradigm, profoundly impact the cognitive outcomes of their offspring [56]. Several early studies revealed that animals subjected to sporadic maternal care exhibit progressive cognitive deficits in adulthood. They also display impaired hippocampal long-term potentiation (a molecular basis of learning and memory) and structural changes such as dendritic atrophy and synaptic degeneration [56–58]. Furthermore, the survival of newborn neurons in the hippocampus is dramatically reduced in mice exposed to LBN from postnatal day 2 to day 9. These alterations are associated with aberrant cognitive performance. However, no changes in cell proliferation and neuronal differentiation were observed [59].

In light of the evidence that early traumatic experiences increase the vulnerability to mental illness in adulthood, increasing research interest has been focused on the consequences of traumatic experiences in early life. Chronic exposure to unavoidable plantar electroshock in the early post-weaning period leads to impaired spatial memory in adulthood, as evidenced by poor performance in the Y-maze or Morris water maze [44, 60]. Additionally, rats exposed to a single platform and acute swimming stress during adolescence also show inferior cognitive performance in adulthood, indicating that even relatively brief stress experiences early in life might exert profound, long-lasting effects on cognitive health [41, 61]. Overall, these data from animal models provide new insights into the crosstalk between ELA and cognitive impairment later in life.

#### Studies in humans

The connection between ELA and neurological consequences has been an active area of research since the discovery that adverse childhood experiences are positively linked to poor health outcomes later in life [16]. Numerous clinical studies have identified ELA as a potential risk factor for cognitive impairment (Table 2). These studies primarily focused on child neglect, physical abuse, and parental separation, thus providing an important context for understanding this health concern.

**Table 2:** Clinical data on ELA and cognitive impairment

Type of study	Subjects	Stressors	Outcome measurement	Cognitive outcome	References
Cohort study	13 subjects with ELA, average age, 13(± 2.58) years of age; 21 healthy subjects, average age, 13 (± 1.96) years of age	Neglect, maltreatment, and unstable early environments	The change task (a variant of the stop-signal task)	Cognitive control ↓	[67]
Cohort Study	93 male subjects experienced child separation; 186 male subjects without child separation	Parental separation	The Finnish Defense Forces Basic Intellectual Ability Test	At 20 years: verbal and arithmetic cognitive ability↓; at 70 years: verbal, visuospatial, arithmetic, and general cognitive ability↓	[68]
Cross-sectional study	64 subjects with major depressive disorder and 65 non-depressed controls, 20 to 50 years of age	Assessed by the self-reported Early Life Stress Questionnaire	Composite neuropsychological measures	Working memory and processing speed↓	[74]
Longitudinal study	9942 subjects	Low childhood socioeconomic status, lack of friends, childhood parental mental health problems, and poor parent-child relationships	Orientation and calculation, immediate memory, and delayed memory test	↓	[72]
Longitudinal study	5000 subjects	Domestic violence, physical cruelty, emotional cruelty, harsh parenting, and poly-victimization	The ‘triangles’ social cognition assessment (mean subject age: 13.75 years) and IQ assessment (mean subject age: 8 years)	General cognition↓	[23]
Cross-sectional study	215 undergraduate students, average age = 19.1, 295 community subjects, average age = 36.24	Assessed by the self-reported adverse childhood experiences scale	Wisconsin Card Sorting Test	Cognitive flexibility↓	[75]
Longitudinal study	12,288 subjects, 18 to 42 years of age	Physical abuse and neglect	The Rey Auditory-Verbal Learning Test and Digit-Span Backward Task	Verbal memory and working memory↓	[69]
Longitudinal study	11,475 subjects, average age, 45 years	Low childhood family socioeconomic status and poor childhood social relationships	Telephone interview of cognitive status, word recall, and figure drawing	↓	[73]

*ELA* early life adversity

The symbol “↓” represents a decrease

Parent-child coregulation is a dynamic process that involves mutual influence and coordination of emotional, behavioral, and physiological states between parents and their children [62]. Early coregulation is critical for the healthy development of children across multiple domains, including emotional and cognitive functioning [63, 64]. The importance of coregulation has been further highlighted by a longitudinal study which indicates that a secure infant-caregiver attachment during infancy may predict adult competence of children, including educational attainment, occupational success, and social functioning [65]. Intriguingly, a longitudinal study has demonstrated that positive mother–child interactions in kindergarten are associated with an increased likelihood of high school graduation and, for some students, a better academic performance [66].

One study examined the cognitive performances of adolescents with a documented history of ELA. Results showed that individuals who experienced child neglect, maltreatment, and unstable early environments exhibited poorer performance in the change task, a task designed to specifically assess cognitive control [67]. Similarly, a Helsinki birth cohort study investigated the ongoing effects of parental separation. At the age of 20 years, men who were separated from their parents during World War II scored lower in verbal, visuospatial, and general cognitive reasoning, and later at the age of 70, they exhibited lower scores in all tests in comparison to non-separated subjects [68]. Of note, a more extended separation period is associated with poorer overall cognitive performances. Several longitudinal studies have assessed how childhood neglect and maltreatment can influence cognitive outcomes later in life, revealing a remarkable connection between childhood neglect/maltreatment and lower general cognition or poorer working memory [23, 69]. Given that different forms of ELA often co-occur in some individuals, the effect of poly-victimization, which refers to experiencing multiple forms of victimization during a certain period, has been further analyzed. Those exposed to multiple types of ELA including physical/emotional abuse, harsh parenting, and domestic violence, experience more severe detrimental effects [23]. The adverse effects of ELA are further supported by the Romanian orphanage studies performed by many groups, which showed that childhood neglect/deprivation led to long-lasting effects on cognitive and emotional development [70, 71]. Children raised in institutions experienced severe deprivation (lack of individualized care and nurturing environment) early in life, and they exhibited lower executive functioning and increased diagnostic susceptibility to psychopathology compared to their peers who were never institutionalized [70]. Early childhood deprivation is associated with structural changes of the brain in adulthood. Adoptees who experienced early deprivation exhibited smaller total-brain volumes compared to nondeprived adoptees, which may be linked to lower intelligence quotient and higher levels of attention-deficit/hyperactivity disorder symptoms [71].

The effects of other types of ELA on cognition have also been examined. A nationally representative longitudinal cohort study analyzed the cumulative effects of multiple childhood adversities on cognitive decline in later life. The results of this study are profound: poor parent-child relationships, lack of friends, childhood parental mental health issues, and low socioeconomic status all negatively affected cognitive function among the middle-aged and elderly populations [72]. These conclusions are further supported by a longitudinal study demonstrating that low family socioeconomic status and poor social relationships in childhood are positively associated with the risk of mild cognitive impairment [73]. To gain more insights into the relationship between the number of ELA exposures and negative outcome expectations, scales with high internal consistency, validity, and test-retest reliability have been developed and employed in several cross-sectional studies. These studies showed that greater ELA exposure is associated with compromised cognitive flexibility, processing speed, and working memory [74, 75]. Moreover, these negative consequences may be amplified in subjects with depression, as evidenced by smaller orbitofrontal cortex and hippocampal volumes compared to never-depressed individuals [74]. Taken together, these human studies provide moderate evidence that ELA exposure is a risk factor for developing cognitive impairment later in life.

### ELA and AD

#### Studies in animals

Numerous experimental AD models have been developed in rodents, exhibiting multiple key aspects of human AD pathology. The potential involvement of ELA in AD has been studied in rodent models (Table 3). APPswe/PS1dE9 mice exposed to LBN displayed aggravated Aβ plaque load at 10 months of age accompanied by increased glial activation and inflammatory signals in the hippocampus [76, 77]. LBN also increased the hippocampal levels of Aβ40 and Aβ42 and elevated the level of β-site APP-cleaving enzyme 1 (BACE1), a key rate-limiting enzyme for Aβ processing and production, in this mouse model [78]. In addition, synaptic damage and cognitive impairment are exacerbated by LBN exposure [78, 79].

**Table 3:** Animal studies on ELA and Alzheimer’s disease

ELA model	Species/sex	Period of exposure	Time of outcome measurement	Cognitive outcome/AD pathology	References
MS	Wistar rats/male	PND 2–21	Adulthood	Cognitive outcome↓ AD pathology↑	[82]
LBN	APPswe/PS1dE9 mice/male	PND 2–9	4 and 10 months of age	At 10 months of age, AD pathology↑	[76]
MS	APPswe/PS1dE9 mice/male	PND 2–21	9 months of age	Cognitive outcome↓ AD pathology↑	[80]
LBN	APPswe/PS1dE9 mice/male	PND 2–9	6 and 9 months of age	Cognitive outcome↓ AD pathology↑	[78]
LBN	APPswe/PS1dE9 mice/male	PND 2–9	6 and 12 months of age	Cognitive outcome↓ AD pathology↑	[79]
LBN	APPswe/PS1dE9 mice/male	PND 2–9	4 and 10 months of age	AD pathology↑	[77]
MS	App^NL−G−F^ mice/male	PND 2–15	1.5 and 4 months of age	Cognitive outcome↓ AD pathology↑	[81]

*AD* Alzheimer’s disease; *ELA* early life adversity; *LBN* limited bedding and nesting conditions; *MS* maternal separation; *PND* postnatal day

The symbol “↓” represents a decrease, and “↑” indicates an increase

Moreover, using different transgenic mouse models of AD, several groups have investigated the effects of maternal separation on AD disease progression. Hui et al. showed that chronic maternal separation worsened cognitive deficits and led to increased Aβ plaque formation and neural damage in adult APPswe/PS1dE9 mice [80]. Tanaka and colleagues focused on vascular pathological changes following maternal separation [81]. In their study, amyloid precursor protein (APP) wild-type and heterozygous APP mutant (*App*^NL−G−F/wt^) mice displayed narrowed vessels in the prefrontal cortex with decreased capillary pericyte coverage and blood-brain barrier (BBB) disruption. Further analysis revealed that these deficits resulted from microglial activation. In addition, maternally separated *App*^NL−G−F/wt^ mice exhibited exacerbated cognitive impairment at four months of age [81]. More importantly, maternal separation has also been reported to induce Aβ formation in animals without an AD background. Martisova et al. exposed Wistar rats to three weeks of maternal separation, and found increases in Aβ40 and Aβ42 levels as well as increased expression of BACE1 in the hippocampus of these animals, along with profound cognitive deficits. These results suggest that ELA increases the risk of AD-like pathology development even in the absence of AD risk genes [82]. Furthermore, the effects of ELA exposure on AD pathology may occur through several common pathogenic mechanisms, perhaps differing by the stressor source.

#### Studies in humans

Multiple cross-sectional and longitudinal studies have documented the tight association between ELA and increased risk of AD or other dementias. Given that early parental death has profound implications for adult health outcomes, a longitudinal study conducted by Norton and colleagues analyzed the connection between this form of ELA and AD onset [83]. Their study included 4108 subjects, aged 65–105, with a mean follow-up of 18 months. Logistic regression analysis revealed a higher number of confirmed cases of AD within the 18-month follow-up in the group experiencing parental death in childhood [83]. Similarly, a cross-sectional study conducted in the Australian community used the self-reported childhood trauma questionnaire (CTQ) to evaluate the prevalence of ELA in older people. Based on the National Institute of Aging/Alzheimer’s disease Association workgroup criteria for AD diagnosis, the study showed that individuals with higher CTQ scores were more likely to receive a diagnosis of AD [24]. In addition, a longitudinal study investigated the association of childhood stress with late-life dementia and AD in 2682 male subjects. After adjustment via Cox regression analysis, a higher prevalence of AD was revealed among those who lived in custody or an orphanage, experienced an early-life crisis, had problems with teachers, or emigrated because of war during childhood [84].

In light of the increasing prevalence of dementia in Japan, several cohort studies have been conducted in Japan to address the crosstalk between ELA and dementia prevalence [85, 86], using the adverse childhood experience questionnaire, which covers family violence, physical and psychological abuse, neglect, parental death, parental divorce, and parental mental illness. Intriguingly, increased numbers of clinically confirmed dementia cases were reported within a 3-year follow-up period in participants with 3 or more adverse childhood experiences [85]. Further investigations indicated that the individual-level social capital score might be a variable for this vulnerability. After stratification by social capital score, this increased dementia risk was only observed in participants with low social capital [86]. More recently, several cross-sectional studies addressing ELA and cognitive outcomes have been performed in the USA. Among both older individuals or former National Football League players, those who experienced more than 4 ELA events had higher rates of positive dementia screening than those without a history of ELA [87, 88].

Taken together, these results indicate that all types of ELA confer an increased risk of developing dementia/AD, despite the widely varying ELA experiences among individuals. The studies mentioned above are summarized in Table 4.

**Table 4:** Clinical data on ELA, Alzheimer’s disease, and dementia

Type of study design	Subjects	Stressors	Follow-up	Diagnosis of AD/other dementias	References
Longitudinal study	4108 subjects, 65–105 years of age	Parental death	18 months	↑	[83]
Cross-sectional study	296 subjects, 66.1 (± 5.8) years of age	Assessed by the childhood trauma questionnaire	Not applicable	↑	[24]
Longitudinal study	2682 men, 42 and 61 years of age	Living in custody or an orphanage, experience of crisis in childhood, having problems with teachers, and emigrating because of war.	Not applicable	↑	[84]
Cohort study	17,412 subjects, 65 years of age or older	Assessed by the adverse childhood experience questionnaire	3 years	↑	[85]
Cohort study	16,821 subjects, 65 years of age or older	Assessed by the adverse childhood experience questionnaire	3 years	↑	[86]
Cross-sectional study	1488 subjects, 65 years of age or older	Assessed by the adverse childhood experience questionnaire	Not applicable	↑	[87]
Cross-sectional study	1755 men, 57.2 (± 13.5) years of age	Assessed by the adverse childhood experiences questionnaire	Not applicable	↑	[88]

*AD* Alzheimer’s disease; *ELA* early life adversity

The symbol “↑” represents an increased diagnosis of AD/other dementias

### Potential mechanisms linking ELA to cognitive impairment and AD

**Fig. 1:**
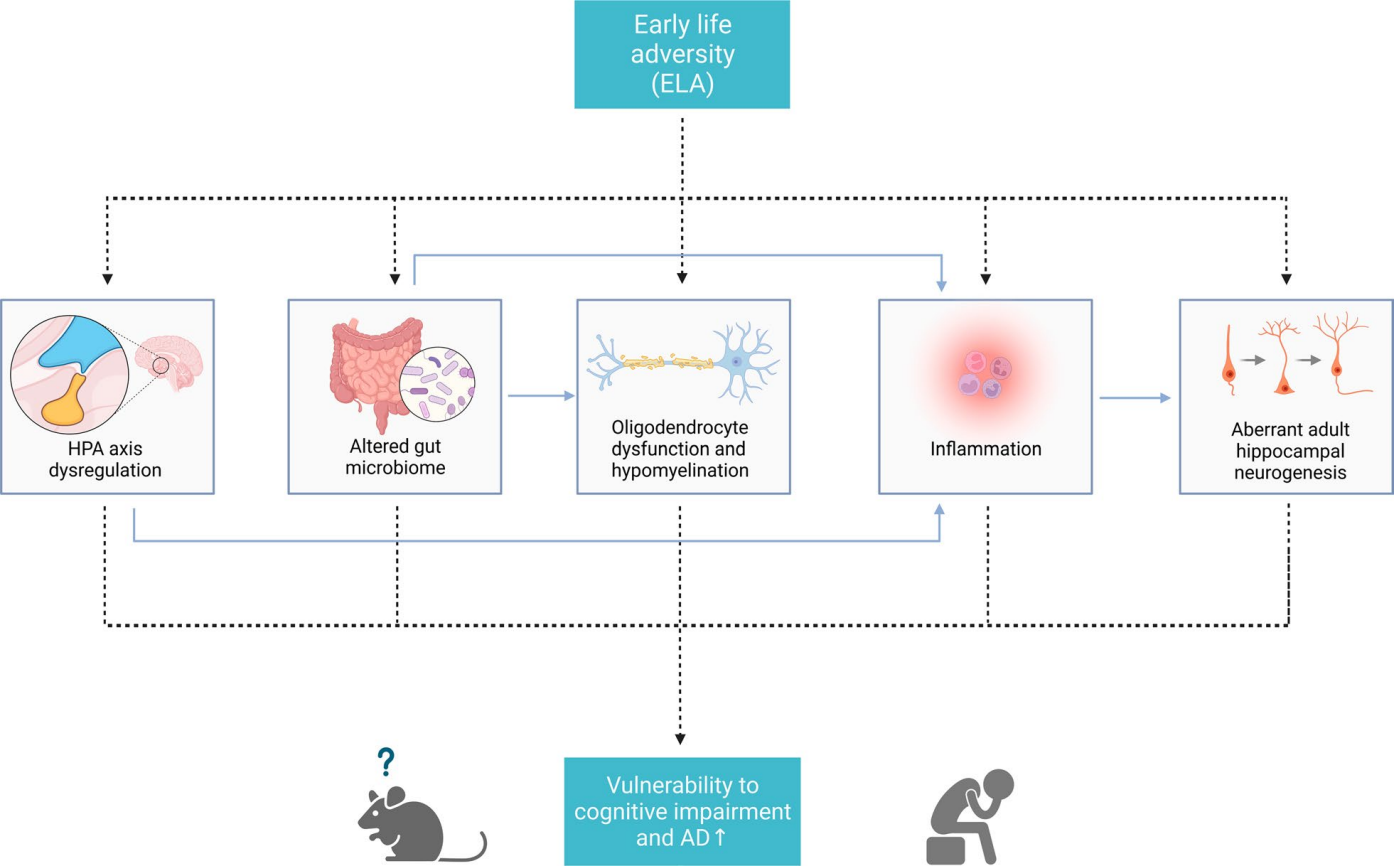
The potential mechanisms underlying increased vulnerability to cognitive impairment and AD following ELA. ELA exposure can induce multiple physiological and pathological processes. Several key mechanisms may be implicated in the increased vulnerability to cognitive impairment and AD in later life, including HPA axis dysregulation, altered gut microbiome, oligodendrocyte dysfunction and hypomyelination, inflammation, and aberrant adult hippocampal neurogenesis. Furthermore, these pathological processes may interact with each other and collectively contribute to adverse neurological outcomes. HPA, Hypothalamus-pituitary-adrenal

Understanding the mechanisms by which ELA influences the vulnerability to cognitive impairment and AD later in life can facilitate the development of preventive interventions and allow their timely application. Despite different stressors among studies, available evidence indicates that different ELAs can affect cognitive outcomes through shared mechanisms (Fig. 1).

#### Hypothalamus-pituitary-adrenal (HPA) axis dysregulation

The HPA axis is one of the major stress response systems in human bodies, and is responsible for the production of stress hormone glucocorticoids, including cortisol and corticosterone [89]. A well-functioning and dynamic HPA axis is essential for coping with stresses in daily life. Chronic stress, however, can disrupt its negative feedback system, leading to chronic activation of the HPA axis and elevated serum cortisol levels [90]. Dysregulated HPA axis and aberrant circulating cortisol levels have been associated with cognitive impairment and AD [91, 92]. Experiments in laboratory animals have shown that glucocorticoid administration can aggravate Aβ formation by increasing levels of APP and BACE [93]. Furthermore, elevated glucocorticoid also exacerbates the development of neurofibrillary tangles [93]. Conversely, mifepristone, an antagonist for glucocorticoid, has been extensively reported to alleviate AD pathology and cognitive decline [94, 95]. Therefore, an aberrant HPA axis may play a key role in the progression of cognitive impairment.

The HPA axis is highly plastic in early life. Notably, the HPA axis of infants is particularly sensitive to parental care, and absence of maternal care could negatively affect the HPA function of their offspring. Experiments performed on zebra finches revealed that maternally deprived offspring were hyper-responsive to stressors, exhibiting higher corticosterone concentrations and altered corticosteroid receptors in the brain [96]. In addition, prenatal stress can have a long-lasting impact on the reactivity of the HPA axis and the cognitive performance of the offspring [97, 98]. Molenaar et al. [98] found that maternal stress during pregnancy (e.g. long-lasting difficulties, stressful life events, family functioning, and pregnancy-related stress) was associated with higher child hair cortisone levels. Animal studies in rats showed that the offspring of prenatally stressed dams exhibited higher anxiety levels and elevated glucocorticoid levels in response to stressors [99].

The early postnatal environment is another critical factor influencing the HPA axis reactivity and sensitivity to stressors later in life. Mice exposed to LBN display hyperreactivity of the HPA axis and alterations of neuroendocrine stress responsiveness [100]. Moreover, maternal separation in rats induced changes of the HPA axis reactivity and increased stress hormone levels, accompanied by poorer cognitive performances in the Morris water maze and the novel object recognition test [101]. Strikingly, these deficits were entirely reversed by both mifepristone and β-adrenoceptor antagonist propranolol. These findings were supported by another study showing that LBN aggravated AD progression through activation of the HPA axis [78]. Intriguingly, short-term blockade of glucocorticoid receptors at 12 months of age was able to impede the accelerated progression of AD pathology due to ELA [78]. Importantly, a study by Kumsta et al. [102] reported that the effects of early-life institutional deprivation in Romanian orphanages can persist into adulthood. Individuals who experienced early life institutional deprivation showed HPA axis dysregulation in adulthood, including blunted cortisol responses to stress and alterations in cortisol metabolism [102].

Therefore, it is not surprising that ELA can cause aberrant HPA axis reactivity, potentially persisting lifelong and increasing susceptibility to cognitive impairment and AD. In this regard, interventions targeting the HPA axis may hold promise in alleviating the complex sequellae of adverse consequences of ELA.

#### Altered gut microbiome

A growing body of work over the last several years suggests that the gut microbiota impacts brain function. There are bidirectional interactions between the intestine and the CNS, known as the brain-gut-microbiome axis [103, 104]. To date, several mechanisms have been reported for the interactions between the gut microbiome and the CNS, including the immune system, the vagus nerve, the enteric nervous system, and the microbial-derived intermediates (e.g., short-chain fatty acids, secondary bile acids, and tryptophan metabolites) [103, 105]. Increasing evidence indicates that the gut microbiota plays a crucial role in neuroimmune signaling. Accordingly, absence of normal gut microbiota could directly disrupt CNS neurotransmission [106]. Gut microbiota is highly sensitive to the early environment, and colonization of microbiota in early life is increasingly connected to the host immune system [107]. Therefore, early life experiences may exert lasting effects on gut microbiota and the development of the host immune system through a “window of opportunity”.

Recent studies have reported that abnormalities in the gut microbiome are implicated in AD pathogenesis. The gut microbiota of patients with AD is strikingly different from that of healthy individuals [108, 109]. Moreover, people with mild cognitive impairment show similar alterations of the gut microbiota as AD patients [110, 111]. Experimental animal studies further support these findings. Gut microbiome analysis using 16S rRNA gene sequencing identified and characterized different compositions of the gut microbiota in transgenic AD animals and healthy controls [112–114]. Interestingly, cerebral amyloid plaques and neurofibrillary tangle pathology were significantly reduced in germ-free 3×Tg AD mice compared to mice with typical gut microbiota [112]. Microbiota transplantation from healthy animals also alleviated amyloid burden and tau pathology [113]. Conversely, gut microbiota from AD individuals aggravated AD disease progression and impaired cognitive function in normal animals [112, 115]. The reshaping of the gut microbiome using probiotics or nutrients has shown promise for AD treatment in several studies [116–118]. Taken together, these findings point toward the role of altered gut microbiota in driving cognitive impairment.

It has been demonstrated that stress induces gut microbiota alterations and disrupts the intestinal barrier integrity [119]. Recently, the effects of ELA on the brain-gut-microbiome axis have gained increasing attention. In young adulthood, maternally separated animals (from PND 2 to 12) showed an altered systemic immune response and increased visceral sensation, along with an alteration of the fecal microbiota [120]. Further studies revealed that alterations in *Butyricimonas*, *Butyricicoccus*, and *Corynebacterium* populations partially mediated the ELA-induced visceral hypersensitivity [121]. Using a multi-hit ELA model, Rincel et al. revealed sex-dependent gut dysbiosis in C3H/HeN mice. Male mice exposed to ELA displayed a significantly lower abundance of *Lachnospiraceae* and *Porphyromonadaceae*, accompanied by an increased abundance of *Bacteroides*, *Lactobacillus*, *Porphyromonas*, *Alloprevotella*, and *Firmicutes* genera. Female mice, on the other hand, only showed changes in *Lactobacillus* and *Mucispirillum* genera [122]. These results resemble gut microbiome alterations observed in early AD [123–125]. In addition to these changes, elevated levels of pro-inflammatory cytokines were observed in the colons of ELA-exposed animals [126]. Similarly, early weaning stress in piglets resulted in increased gut barrier permeability and diminished expression of gut epithelial tight junction proteins, along with higher levels of pro-inflammatory cytokines [127]. Notably, significant gut microbiome alterations have been observed in human subjects with a history of ELA [128, 129].

Taken together, these findings suggest that alterations in the brain-gut-microbiome axis play a role in ELA-induced cognitive impairment. However, the mechanisms by which ELA affects the brain-gut-microbiome axis and the exact microbiota that contributes to these adverse neurological consequences remain to be determined.

#### Oligodendrocyte dysfunction and hypomyelination

Another potential mechanism that links ELA to cognitive impairment and AD is oligodendrocyte dysfunction and hypomyelination. The myelin sheath is a multi-layered structure wrapping around the axon [130]. In the CNS, myelin sheaths are formed by mature oligodendrocytes through a process called myelination [130]. Myelination of axons is essential for normal CNS function in vertebrates. Intact myelin sheaths contribute to the conduction velocity of nerve impulses and provide trophic support for neurons and their axons [130, 131]. Intriguingly, emerging evidence highlights the importance of myelin in retaining cognition [132, 133]. Extensive myelin loss has been reported in AD patients and rodent models of AD, and enhancing myelin renewal rescues cognitive impairment in experimental animals [133, 134]. Moreover, depletion of oligodendrocytes or compromised oligodendrogenesis leads to cognitive dysfunction in normal animals [135, 136], whereas enhanced myelination can rescue age-related cognitive deficits [133]. All these findings imply that dysfunctional oligodendrocytes may directly contribute to cognitive dysfunction.

Stress has been identified as a trigger for oligodendrocyte dysfunction and hypomyelination [137]. Animals exposed to chronic stress show aberrant oligodendrocyte differentiation, hypomyelination, and oligodendroglial apoptosis in the prefrontal cortex and hippocampus, two brain areas involved in cognitive processing [138–140]. Furthermore, neuroimaging and postmortem histopathological studies also revealed compromised oligodendrocyte/myelin in individuals who experienced ELA. Postmortem brain samples from individuals with a history of child abuse showed decreased myelin thickness and an aberrant myelin-related transcriptional program [141]. A slower rate of myelin growth was also reported in those who experienced early-life economic disadvantage [142].

Animal studies have provided greater insights into the correlation between ELA, oligodendrocyte dysfunction/hypomyelination, and cognitive impairment. Bordner et al. reported a novel model to mimic early life neglect [143]. They exposed mice to neonatal maternal separation and early weaning, and conducted functional genomic and proteomic analyses after the animals reached adulthood. The results revealed significant dysregulations of myelin-related proteins and genes, including decreased expression of MBP, MAG, MOG, and PLP mRNA [143]. These findings were further supported by studies in rats [144, 145]. Rats separated from their dam during the first three postnatal weeks exhibited pronounced demyelination and aberrant oligodendrocyte differentiation at adulthood, accompanied by notable cognitive deficits [144]. Furthermore, inhibition of the Wnt signaling, which negatively regulates oligodendrocyte development, rescued these deficits [144]. To gain more insights into the abnormal oligodendrocyte processes resulting from ELA, Teissier and colleagues investigated oligodendrocyte changes in both early postnatal and adult stages after ELA induction in mice, and showed different patterns of variations [146]. Specifically, a precocious oligodendrocyte differentiation was observed in the prefrontal cortex immediately after maternal separation. However, depletion of the oligodendrocyte progenitor pool and compromised oligodendrocyte differentiation were observed in adulthood. Further mechanistic studies indicated that ELA decreased neuronal excitability, resulting in premature differentiation of oligodendrocytes and depletion of the oligodendrocyte precursor pool [146]. More importantly, chemogenetic activation of neuronal excitability normalized these alterations in ELA-exposed animals and mitigated memory impairment [146]. These studies suggest that ELA significantly impacts oligodendrocyte physiology, and dysfunctional oligodendrocytes and hypomyelination may contribute to subsequent cognitive deficits. Nevertheless, whether ELA also directly exacerbates myelin loss in the context of AD remains largely unclear. Further research is needed to address this issue.

#### Inflammation

As an integral part of innate immunity, the inflammatory response is the body’s self-protection mechanism to eliminate pathogens and protect against pathogen invasion. However, sustained or chronic inflammation can contribute to the progression of multiple diseases and neurodegeneration [147, 148]. Inflammation, particularly neuroinflammation, has been increasingly linked with cognitive impairment and neurodegeneration, persisting throughout the course of the diseases. Indeed, inflammation is a prominent feature of mild cognitive impairment and AD [149, 150]; elevated pro-inflammatory cytokines and chemokines have frequently been observed in the cerebrospinal fluid from patients with cognitive impairment or AD and are associated with amyloid and tau pathology [151, 152]. A meta-analysis involving 28 studies also revealed higher levels of inflammatory markers throughout the brain in people with cognitive impairment and confirmed AD cases [150]. In addition, exposure to acute inflammation aggravated the progression of AD in a mouse model of AD, which was accompanied by significant increases in inflammatory cytokines and chemokines in the brain [153]. Notably, loss of the NLR family pyrin domain containing 3 (NLRP3), an inflammasome implicated in multiple neurological diseases, prevented the development of AD-related pathology. In contrast, injection of fibrillar amyloid-beta-containing brain homogenate from AD animals induced AD pathology in an NLRP3-dependent manner [154]. Therefore, the development of inflammation could be a pivotal contributor to cognitive impairment.

Importantly, exposure to stress conditions can also trigger chronic inflammation. Acute electric foot shock has been shown to trigger NLRP3 inflammasome activation in the hippocampus and compromise fear memory and synaptic protein expression [155]. Of note, these deficits were not observed in Nlrp3-/- mice or animals treated with an NLRP3 inhibitor, suggesting the critical involvement of NLRP3 inflammasome in stress-driven inflammation [155]. Similarly, the expression of pro-inflammatory factors was elevated in animals exposed to neonatal maternal separation, which was accompanied by memory impairment [156, 157]. Furthermore, in APP/PS1 mice, LBN exposure led to lasting alterations in inflammation response, including increased hippocampal interleukin-1β expression and microglial activation, coinciding with accelerated disease progression [76].

Stress, including ELA, also leads to significant alterations of the neuroimmune profile. Microglia, the primary resident immune cells in the brain, are involved in phagocytosis and can release cytokines and neurotrophic factors [158]. Typically, microglia polarize into one of two different subtypes: pro-inflammatory (M1 phenotype) and anti-inflammatory (M2 phenotype) [159]. Both acute and chronic stress can trigger M1-phenotype microglial activation, causing the release of pro-inflammatory cytokines [76, 160, 161]. For instance, electric foot shocks and intermittent noise have been reported to induce microglial M1 polarization [136]. At four months of age, APPswe/PS1dE mice exposed to LBN (from PND 2 to 9) showed microglial activation and elevated levels of pro-inflammatory factors, along with an altered microglia response to Aβ neuropathology [76].

The activation of microglia and elevated pro-inflammatory factors were also observed in rats exposed to maternal separation [162]. Furthermore, activation of the stress system usually mediates alterations of the BBB permeability, which may further exacerbate the flow of peripheral inflammatory factors into the brain [163, 164]. Hence, chronic inflammation may be an important player connecting ELA and cognitive impairment/AD. However, further research is warranted to gain an in-depth understanding of the mechanisms by which ELA triggers chronic systemic inflammation and neuroinflammation. Notably, in addition to the pro-inflammatory activation of microglia, ELA has been reported to impair microglial function. Exposure to LBN resulted in compromised microglial process dynamics and deficits in microglial engulfment of synapses, leading to excess excitatory synapses in the hypothalamus and aberrant behavioral stress response [165]. Conversely, selective activation of microglia in early life has been shown to rescue these deficits. Therefore, microglial actions during development may also contribute to the adverse consequences of ELA [165].

#### Aberrant adult hippocampal neurogenesis (AHN)

The mammalian hippocampus, in particular the dentate gyrus (DG), is one of the few brain regions capable of generating new neurons throughout life. This process is known as AHN [166]. As a well-organized process, AHN involves the proliferation, migration, differentiation, and maturation of adult neural progenitor cells and has been implicated in many cognitive processes [166, 167]. Altered AHN has been reported in AD and may represent an early critical event in disease progression [168, 169]. Using state-of-the-art tissue processing methods, Moreno-Jimenez et al. observed immature neurons with variable degrees of maturation in the DG from neurologically healthy human subjects; however, the number and maturation of these neurons progressively declined alongside AD development [169]. Additionally, AHN depletion exacerbated cognitive deficits in AD and contributed to cognitive impairment in normal individuals [170, 171]. Conversely, pharmacological induction of AHN improved cognitive function in a mouse model of AD, suggesting a critical role of AHN in supporting cognitive function [172].

Importantly, AHN is strongly affected by early life experiences. The effects of ELA on AHN were first reported by Mirescu et al. in 2004. In their study, rat pups were subjected to maternal separation (3 h each day, from PND 1 to 14), and alterations in AHN were detected at adulthood. Strikingly, cell proliferation and immature neuron generation were decreased in the DG of maternally separated animals [173]. In addition, this compromised AHN was further demonstrated to involve a corticosteroid-dependent mechanism, as it could be reversed by lowering the corticosterone level. Similarly, ELA accelerates age-related AHN alterations. Compared to age-matched animals, rats that experienced neonatal maternal separation exhibited diminished dorsal hippocampal neurogenesis in the young adult period. These changes were more profound at 10 months of age, as evidenced by decreased neurogenesis in both the dorsal and the ventral hippocampus [174].

Several studies have further investigated the mechanisms underlying ELA-induced aberrant AHN [59, 175, 176]. Maternally separated animals showed enhanced hippocampal neurogenesis during the postnatal period and young adulthood, whereas compromised AHN was subsequently observed in middle age, compared to age-matched controls [175]. Another study further supported these results, reporting that LBN on PND 2–9 increased the proliferation and differentiation of neurons in the DG at PND 9 [59]. However, DG volume and survival of adult-born neurons were reduced at age of 5 months. Intriguingly, a reduction in neural stem cells was observed in the DG of adult mice that experienced LBN-induced ELA on PND 3–10 [176]. Hence, ELA-induced early progenitor pool depletion may underlie the aberrant AHN, thereby increasing susceptibility to cognitive impairment/AD in later life.

### Potential crosstalk between different pathological mechanisms

Although we proposed several mechanisms to explain increased vulnerability to cognitive impairment/AD following ELA exposure, the adverse neurological consequences of ELA may not result from a single mechanism. Indeed, these mechanisms likely interact with each other and collectively contribute to the onset of cognitive impairment/AD later in life. For instance, evidence has shown that acute administration of glucocorticoids prior to lipopolysaccharide injection sensitizes both peripheral and central inflammatory responses to the immune challenge [177], and potentiate the pro-inflammatory response of hippocampal microglia to lipopolysaccharide [177]. These findings raise the possibility that the higher level of stress hormone glucocorticoid resulting from dysfunctional HPA axis may intensify inflammation, which could form a vicious feedback loop and lead to adverse neurological outcomes. There has also been accumulating evidence for the association between inflammation and neurogenesis. Pro-inflammatory cytokines produced peripherally can communicate with the brain and activate resident microglia, which subsequently release additional pro-inflammatory cytokines, ultimately impairing hippocampal neurogenesis [178, 179]. Moreover, the gut microbiome can also affect microglia and myelination in the brain [180, 181]. Disruption of the gut microbiome has been found to worsen the activation of microglia and neuroinflammation in mice, while administration of fecal microbiota transplants from healthy mice mitigates neuroinflammation [182]. Recent investigations have also suggested a connection between gut microbiome and myelination. Compared to conventionally raised mice, germ-free mice exhibited up-regulation of genes linked to myelination and myelin plasticity in the prefrontal cortex, concomitant with increased myelin sheath thickness [181]. Hence, healthy gut microbiomes may have a profound effect on oligodendrocyte development and myelin formation, but the specific intestinal microbes involved in myelination are still poorly understood.

Overall, although there has been a growing interest in the field, further investigations are necessary to ascertain the potential crosstalk between these pathological changes. Such research may provide critical insight into the intervention strategies to prevent the adverse outcomes of ELA.

### Management of ELA and potential interventions

ELA affects children worldwide; unfortunately, therapeutic management options remain limited. Currently, ELA management focuses on preventive strategies. Family-targeted anticipatory guidance effectively fosters positive family functioning, improves parent-child relationships, and reduces child abuse and neglect [183]. Accordingly, several policy changes to improve early living conditions of children have been implemented [184]. Nevertheless, a substantial number of adverse consequences of ELA may not be apparent until later in life. Hence, post-hoc interventions aiming to mitigate the negative consequences of ELA are urgently required. In light of the increasing evidence that ELA is a risk factor for cognitive impairment and AD, interventions should be developed to help reduce the burden of these neurological illnesses.

Intriguingly, several interventions have shown the potential to alleviate cognitive impairment in ELA-exposed experimental animal models. Environmental enrichment involves housing animals in stimulating environments to facilitate improvement of cognitive function [185, 186]. Although the programs of environmental enrichment vary among laboratories, they typically involve the introduction of running wheels, toys, and larger cages. Environmental enrichment has shown beneficial effects in preventing ELA-induced cognitive impairment in several studies [187, 188]. Compared to maternally deprived controls, animals treated with early-life environmental enrichment (from PND 21 to 81) exhibited higher hippocampal expression of BDNF and cognitive improvements in adulthood [189]. Early environmental enrichment also preserved normal AHN and hippocampal-dependent functions [188]. More importantly, even short-term early environmental enrichment (from PND 22 to 34) can mitigate the adverse consequences of ELA, as evidenced by the retention of long-term potentiation and improved cognitive performance [190].

Nutritional supplements have also been reported to improve cognitive outcomes following ELA. Omega-3 fatty acids are essential for brain development and function [191, 192]. Recently, Omega-3 fatty acid supplementation has been suggested as a promising approach for AD prevention [193]. In LBN-induced ELA animal models, an early diet with Omega-3 fatty acids prevented cognitive impairments in adulthood and rescued the survival of newborn hippocampal cells [194]. Similarly, early administration of an Omega-3 fatty acid mixture also alleviated cognitive deficits in rats exposed to neonatal maternal separation [195]. Further studies revealed that an Omega-3 fatty acid mixture also altered gut microbiota composition in maternally separated rats, which was associated with a diminished corticosterone response to acute stress [196]. In addition, probiotic supplementation has shown promising results in improving outcomes after ELA [197, 198]. These observations provide promising new therapeutic avenues for ELA.

Exercise represents a promising, cost-effective intervention to prevent adverse consequences of ELA. Our group and others have reported positive effects of exercise in preventing AD and alleviating cognitive impairment in experimental animal models [199, 200]. Likewise, exercise seems to have beneficial effects on ELA-related pathophysiology. In rodent models, early voluntary exercise attenuated ELA-induced behavioral abnormalities in adulthood, accompanied by normalization of corticosterone level and decreased expression of pro-inflammatory factors [201, 202]. In addition, a study by Park and colleagues found that two weeks of treadmill exercise from PND 21 to 35 significantly ameliorated cognitive impairment induced by maternal separation [203]. Furthermore, our recent work demonstrated that photobiomodulation, a non-invasive therapy, alleviated oligodendrocyte dysfunctions induced by early unpredictable electric foot shock and prevented adverse outcomes, indicating the therapeutic potential of this treatment for ELA [42].

Notably, the therapeutic window is an important consideration when establishing ELA management strategies. Most preclinical studies to date have indicated that many interventions are effective in preventing ELA-induced cognitive impairment when administered early. Unfortunately, later interventions may sometimes be less effective. For instance, exercise, when initiated from 8 months of age, significantly promoted AHN; however, this effect was diminished in ELA-exposed animals [45]. Therefore, early intervention might be particularly important. Lastly, despite these encouraging results, the wide varieties of ELA type and degree make it challenging to establish standardized treatments and univocal guidelines from a clinical perspective. Further studies are thus needed to establish optimal treatment strategies, time windows, and dosages for individuals who have experienced different types of ELA.

## Conclusion

As an environmental factor, ELA has tremendous impacts on brain development and can increase future susceptibility to neurological illnesses. The present review emphasizes ELA as a risk factor for cognitive impairment and AD in later life. ELA may contribute to the pathogenesis of cognitive impairment/AD through multiple pathophysiologic events, with potential crosstalk between mechanisms. Additionally, several interventions, including environmental enrichment, exercise, and nutritional supplements, have shown potentials in mitigating these adverse neurological consequences. Further studies are warranted to unravel the exact mechanisms underlying increased susceptibility to cognitive impairment and AD following ELA. Developing effective early interventions in parallel would help alleviate the disease burdens on individuals, their families, and the public health.

## Data Availability

Not applicable.

## Abbreviations

AD: Alzheimer’s disease
AHN: Adult hippocampal neurogenesis
APP: Amyloid precursor protein
BBB: Blood-brain barrier
BDNF: Brain-derived neurotrophic factor
CTQ: Childhood trauma questionnaire
CNS: Central nervous system
DG: Dentate gyrus
ELA: Early life adversity
LBN: Limited bedding and nesting conditions
BACE1: β-site APP-cleaving enzyme 1
HPA: Hypothalamus-pituitary-adrenal
NLRP3: NLR family pyrin domain containing 3
